# Associated factors of osteoporosis and vascular calcification in patients awaiting kidney transplantation

**DOI:** 10.1007/s11255-023-03606-0

**Published:** 2023-04-24

**Authors:** Junhao Lv, Wenqin Xie, Suya Wang, Yilin Zhu, Yaomin Wang, Ping Zhang, Jianghua Chen

**Affiliations:** 1 Kidney Disease Center, College of Medicine, The First Affiliated Hospital, Zhejiang University, Hangzhou, China; 2 Key Laboratory of Kidney Disease Prevention and Control Technology, Hangzhou, China; 3 National Key Clinical Department of Kidney Diseases, Institute of Nephrology, Zhejiang University, Hangzhou, China; 4 Zhejiang Clinical Research Center of Kidney and Urinary System Disease, Hangzhou, China

**Keywords:** Secondary hyperparathyroidism, Bone turnover markers, Vascular calcification, Osteoporosis

## Abstract

**Introduction:**

Pretransplant osteoporosis and vascular calcification probably increase the risk of fractures and cardiovascular events after kidney transplantation. In the present study, we investigated the related risk factors of osteoporosis and vascular calcification among end-stage renal disease (ESRD) patients awaiting kidney transplantation.

**Methods:**

A total of 221 ESRD patients (age, 43.4 ± 14.3 years; 125 males and 96 females; median dialysis duration, 61.0 m) awaiting kidney transplantation were enrolled in this cross-sectional study. Serum levels of bone turnover markers and intact parathyroid hormone (iPTH) were analyzed from fasting morning blood samples. Dual-energy X-ray absorptiometry was used to measure bone mineral density (BMD). Vascular calcification was evaluated by lateral abdominal radiography and plain radiographic films of the pelvis and hands.

**Results:**

The osteoporosis prevalence was 27.6% in this cohort of kidney transplantation candidates, and the prevalence of vascular calcification was 51.1%. The related factors for osteoporosis and vascular calcification were similar and included older age, longer dialysis duration, parathyroid hyperplasia, and higher levels of iPTH and bone turnover markers. In the multivariable regression model, age and iPTH were independent risk predictors of both vascular calcification and osteoporosis. There were strong, positive correlations between iPTH and all bone turnover markers. The moderate and severe hyperparathyroidism (iPTH 600–1499 pg/ml and iPTH 1500 pg/ml) were related to reduced serum albumin and hemoglobin levels.

**Conclusion:**

The involvement of high iPTH levels in vascular calcification, osteoporosis, and malnutrition indicated the need of treating hyperparathyroidism early in patients awaiting kidney transplantation. Prospective studies are needed to further examine the utility of bone turnover markers.

## Introduction

Mineral and bone disorder (MBD) is a common complication of chronic kidney disease (CKD), with increasing prevalence as CKD progresses. CKD-MBD is characterized by abnormal mineral metabolism, renal osteodystrophy, and/or extraosseous calcification [1]. Renal osteodystrophy is responsible for major morbidity, including fractures, and a deterioration in quality of life [2]. Compared with the general population, the fracture risk among CKD patients steadily increases along with the progression of renal disease [3, 4] and remains high after kidney transplantation [5]. Moreover, pretransplant renal osteodystrophy increased the risk for fractures in renal transplant patients [6].

Extraosseous calcification, especially cardiovascular calcification, predicts subsequent cardiovascular mortality and all-cause mortality in end-stage renal disease (ESRD) patients [7]. A number of studies have demonstrated that osteoporosis is a risk factor for cardiovascular disease [8, 9]. Arterial calcification and osteoporosis are frequently observed in the same subjects and progress in parallel in patients with chronic and end-stage kidney diseases. The links between bone and arterial abnormalities suggest the direct or indirect influence of bone cells and metabolism on the arterial system [10]. Besides, some converging evidences support the existence of a bone–vascular axis after kidney transplantation [11].

Bone biopsy with histomorphometry is the gold standard for the diagnosis and classification of renal osteodystrophy. However, bone biopsy is an invasive procedure and is not practical to perform in all patients. Noninvasive approaches, such as dual energy X-ray absorptiometry (DXA) and biochemical biomarkers of bone turnover, are considered surrogate measures to assess fracture risk [12]. Bone homeostasis involves the actions of resorption and the formation of cells (osteoclasts and osteoblasts, respectively). Bone turnover biomarkers are directly released during the process of bone resorption (e.g., collagen type 1 crosslinked C-telopeptide, CTX) and bone formation (e.g., N-terminal propeptide of type 1 procollagen, PINP, and osteocalcin, OC) [13]. However, the diagnostic and prognostic value of bone turnover markers is still disputed.

Furthermore, the relationship between bone remodeling and vascular calcification is perplexing. The purpose of the present study was to explore the risk factors for osteoporosis and vascular calcification and evaluate their relationship with biochemical markers among ESRD patients awaiting kidney transplantation.

## Methods

### Patients

Enrolled patients were ESRD patients and registered on a kidney transplant waitlist at the First Affiliated Hospital, College of Medicine, Zhejiang University during November 2018 and June 2020. Candidates were excluded if they had previously undergone parathyroidectomy, had primary hyperparathyroidism, had new-onset fracture within 12 months, received bone-affecting treatment (such as bisphosphonate, corticosteroids, calcitonin), or lacked adequate laboratory data. This study was approved by the Ethics Committee of the First Affiliated Hospital, College of Medicine, Zhejiang University (Medical Ethical Committee, No. IIT20210363A), and complied with the principles of the Declaration of Helsinki. All patients provided written informed consent.

### Biochemical measurements

Blood samples were collected from each patient in the morning after an overnight fast. In hemodialysis (HD) patients, blood was collected before the initiation of dialysis. The serum concentrations of intact parathyroid hormone (iPTH), 25 hydroxyvitamin D [25(OH)D], P1NP, OC and CTX were measured using an electrochemiluminescence immunoassay analyzer (Roche Diagnostics GmbH, Mannheim, Germany). Serum alkaline phosphatase (sALP), phosphate, and calcium were analyzed by standardized methods throughout the study period. Serum calcium levels were corrected for serum albumin.

### Bone density and vascular calcification measurements

BMD was measured using DXA (Hologic Inc., Bedford, MA, USA) at the lumbar spine (L2 through L4) and femoral neck. The diagnosis of osteoporosis was determined by the occurrence of a fragility fracture or the World Health Organization (WHO) cutoff value of a T score of ≤ − 2.5 at the spine and femur in patients without fracture. Vascular calcification was evaluated by lateral abdominal radiography and plain radiographic films of the pelvis and hands. The presence of linear calcifications was considered vascular calcification. Echocardiogram was also used to detect the presence or absence of valvular calcification. Parathyroid hyperplasia was identified by ultrasound exam and technetium-99m-sestamibi scintigraphy.

### Statistical analysis

Descriptive data are presented as the mean (standard deviation, SD) for normally distributed variables and as the median (interquartile range) for non-normally distributed variables. Categorical variables are expressed as absolute numbers with percentages. Categorical variables were compared by Chi-square test. The analysis of variance test was performed for the comparison of parameters between groups by the independent-samples *t* test or one-way analysis of variance (one-way ANOVA). The risk factors for osteoporosis and vascular calcification were evaluated by binary logistic regression analysis. Variable with *P* value < 0.05 in the univariate logistic regression analysis was exported to multivariable binary logistic regression analysis. The cutoff values of risk factors were determined by receiver operating characteristic (ROC) curves. A *P* value less than 0.05 was considered statistically significant. SPSS statistical software (version 22; IBM Corporation, Chicago, IL) was used for all calculations.

## Results

### Characteristics of study participants

Two hundred and twenty-one patients were enrolled in the present analysis. The mean age was 43.4 ± 14.3 years; 56.6% were male. The mean body mass index (BMI) was 21.8 ± 3.1 kg/m^2^. In addition, 215 patients (97.3%) had been on dialysis (131 hemodialysis, 84 peritoneal dialysis), and 6 patients were given conservative treatment without dialysis. The median dialysis duration was 61.0 months (range 0–186 months). The median level of iPTH was 852.0 pg/ml (range 113.4–3864.0 pg/ml). Osteoporosis was present in 27.6% (61/221) of patients, parathyroid hyperplasia in 69.7% (154/221) of patients, and vascular calcification in 51.1% (113/221) of patients.

### Factors associated with osteoporosis

The characteristics of ESRD patients categorized by osteoporosis status are shown in Table 1. Patients with osteoporosis were more likely to be older (*p* < 0.001) and female (*p* = 0.01) and to have a longer dialysis duration (*p* < 0.001). The level of serum albumin was lower (*p* = 0.002), and the levels of bone metabolism markers, including intact PTH (*p* < 0.001), sALP (*p* < 0.001), ß-CTX (*p* = 0.01), and P1NP (*p* < 0.001), were significantly higher in osteoporosis patients. Compared to nonosteoporosis patients, the incidence of parathyroid hyperplasia (93.4% vs. 60.6%, *p* < 0.001) and vascular calcification (75.4% vs. 41.9%, *p* < 0.001) was higher in osteoporosis patients.

**Table 1:** Demographics and parameters of mineral metabolism in ESRD patients categorized according to osteoporosis and vascular calcification status

Characteristics	Osteoporosis			Vascular calcification		
	Yes (*n* = 61)	No (*n* = 160)	*P*	Yes (*n* = 113)	No (*n* = 108)	*P*
Age (year)	51.3 ± 11.3	40.4 ± 14.2	< 0.001	52.5 ± 11.6	33.9 ± 10.2	< 0.001
Female (*n*, [%])	35 (57.4%)	61 (38.1%)	0.01	56 (49.6%)	40 (37.0%)	0.06
BMI (kg/m^2^)	21.8 ± 2.9	21.7 ± 3.1	0.9	22.0 ± 2.8	21.5 ± 3.3	0.3
Hemodialysis (*n*, [%])	42 (68.9%)	89 (55.6%)	0.1	73 (64.6%)	58 (53.7%)	0.02
Dialysis duration (mo)	86 [63–114]	41 [5.3–85]	< 0.001	89 [62–115]	12.5 [3.3–55.8]	< 0.001
Hypertension (*n*, [%])	57 (93.4%)	143 (89.4%)	0.4	105 (92.9%)	95 (88.0%)	0.3
Serum albumin (g/L)	38.2 ± 4.6	40.5 ± 5.9	0.002	38.1 ± 5.2	41.7 ± 5.6	< 0.001
Hemoglobin (g/L)	100.7 ± 15.1	103.8 ± 17.2	0.2	101.2 ± 16.6	104.8 ± 16.6	0.1
Serum calcium (mmol/L)	2.3 ± 0.3	2.3 ± 0.2	0.4	2.3 ± 0.2	2.3 ± 0.2	0.6
Phosphates (mmol/L)	2.2 ± 0.6	2.2 ± 0.5	0.9	2.2 ± 0.5	2.1 ± 0.5	0.1
Intact PTH (pg/mL)	1087 [755–1908]	676 [299–1322]	< 0.001	1263 [797–1709]	436 [238–873]	< 0.001
sALP (U/L)	206 [112–324]	113 [69–175]	< 0.001	185 [119–297]	91 [64–135]	< 0.001
ß-CTX (pg/ml)	6000 [4432–6007]	4614 [2970–6001]	0.01	5985 [4492–6009]	3959 [2780–5923]	< 0.001
P1NP (µg/ml)	1201 [896–1204.5]	806.1 [312–1200]	< 0.001	1200 [910–1204]	525 [285–1200]	< 0.001
OC (ng/ml)	264.4 [215–301]	257.8 [209–301]	0.6	277.7 [226–302]	248.7 [193–301]	0.005
25(OH)D (nmol/L)	51.3 [25.2–79.6]	42.4 [25.3–71.1]	0.5	48.3 [26.1–81.7]	42.4 [23.6–61.9]	0.04
Parathyroid hyperplasia (*n*, [%])	57 (93.4%)	97 (60.6%)	< 0.001	101 (89.4%)	53 (49.1%)	< 0.001
Osteoporosis (*n*, [%])	NA	NA		46 (40.7%)	15(13.9%)	< 0.001
Vascular calcification (*n*, [%])	46 (75.4%)	67 (41.9%)	< 0.001	NA	NA	

*PTH* parathyroid hormone, *sALP* serum alkaline phosphatase, *ß-CTX* ß-collagen type 1 crosslinked C-telopeptide, *P1NP* N-terminal propeptide of type 1 procollagen, *OC* osteocalcin, *25(OH)D* 25 hydroxyvitamin D, *NA* not applicable

In multivariable binary logistic regression analysis, osteoporosis was significantly positively associated with age and the level of iPTH (Table 2). According to ROC analysis, the cutoff values of age and iPTH were 47.5 years and 646.7 pg/mL, respectively.

**Table 2:** Association of osteoporosis and vascular calcification with demographic data and biochemical variables determined with multivariate logistic regression analysis (binary) and further ROC analysis

Dependent variable	Independent variables	OR (95% CI)	*p*	AUC	Cutoff value
Osteoporosis	Age (y)	1.039 (1.010, 1.069)	0.008	0.729	47.5
	iPTH (pg/mL)	1.001 (1.000, 1.001)	0.007	0.693	646.7
Vascular calcification	Age (y)	1.162 (1.114, 1.212)	< 0.001	0.876	41.5
	25(OH)D	1.018 (1.006, 1.031)	0.004	0.566 (*p* = 0.089)	NA
	iPTH (pg/mL)	1.001 (1.001, 1.002)	< 0.001	0.783	727.3

*CI* confidence interval, *OR* odds ratio, *AUC* area under curve, *NA* not applicable

### Factors associated with vascular calcification

The clinical and biochemical parameters of the study participants by vascular calcification status are shown in Table 1. Similar to osteoporosis, the ESRD patients with vascular calcification were older (*p* < 0.001), were more likely to be on hemodialysis (*p* = 0.02), had a longer dialysis duration (*p* < 0.001) and had a reduced level of serum albumin (*p* < 0.001). The levels of intact PTH, sALP, ß-CTX, P1NP (*p* < 0.001), OC (*p* = 0.005) and 25(OH)D (*p* = 0.04) were significantly higher in patients with vascular calcification. Compared to patients without vascular calcification, patients with vascular calcification had a higher incidence of parathyroid hyperplasia (89.4% vs. 49.1%, *p* < 0.001) and osteoporosis (40.7% vs. 13.9%, *p* < 0.001).

Vascular calcification was significantly positively associated with age and the levels of 25(OH)D and iPTH according to multivariate analysis (Table 2). The cutoff values of age and iPTH were 41.5 years and 727.3 pg/mL, respectively.

### Changes in biochemical parameters among different iPTH groups

**Fig. 1:**
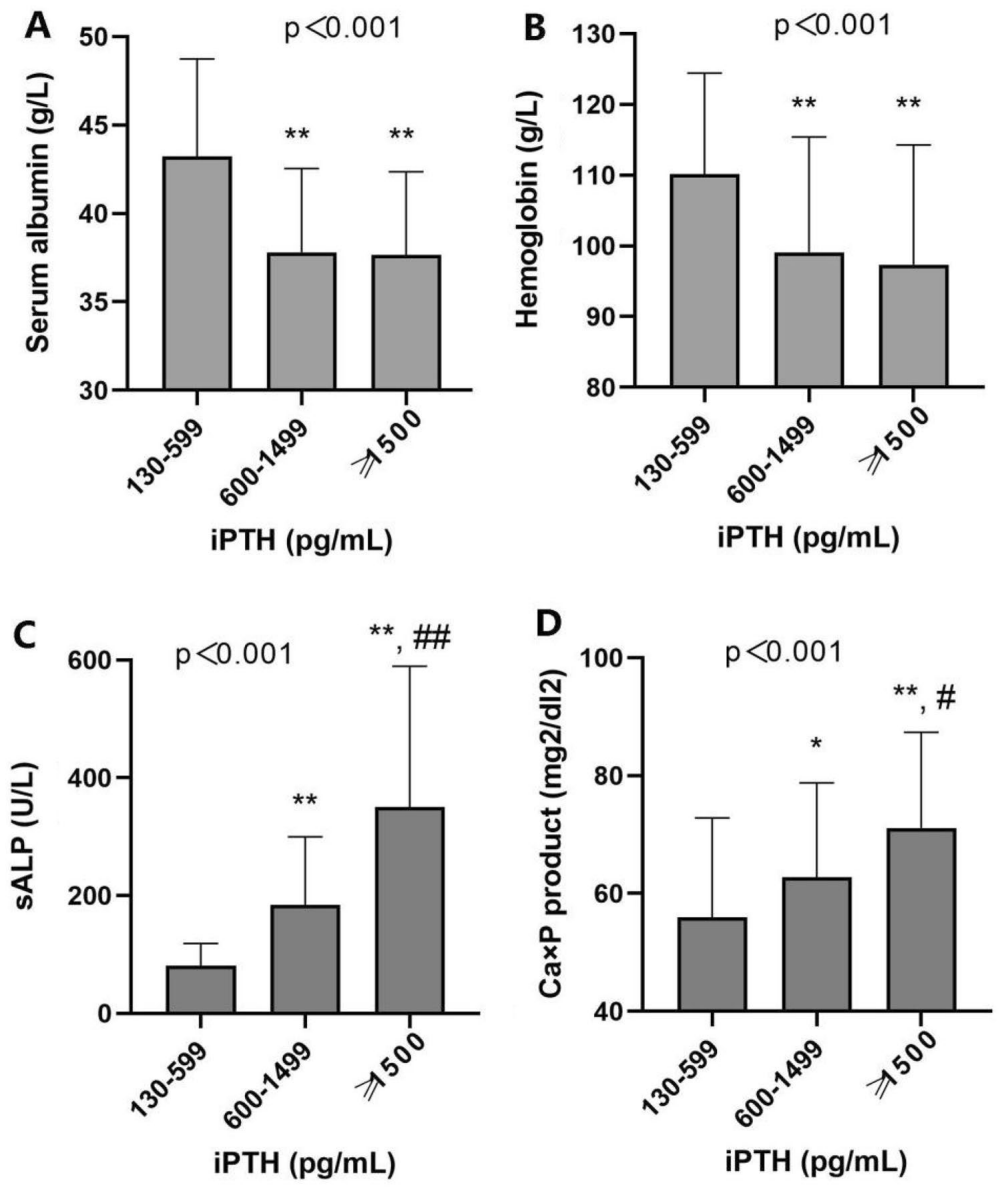
Nutritional and mineral metabolism parameters according to iPTH level. **A** Serum albumin; **B** hemoglobin; **C** serum alkaline phosphatase; and **D** calcium–phosphorus product. *p < 0.05, **p < 0.001 vs. iPTH 200–600; ^#^*p* < 0.05, ^##^*p* < 0.001 vs. iPTH 600–1500

To investigate the changes in biochemical parameters associated with the degree of hyperparathyroidism, study participants were divided into three groups based on iPTH levels: 130–599 pg/ml, 600–1499 pg/ml, and ≥ 1500 pg/ml (Table 3). The dialysis duration was significantly longer among those with iPTH 600–1499 pg/ml and iPTH ≥ 1500 pg/ml groups. Serum albumin and hemoglobin levels decreased as hyperparathyroidism severity increased, whereas serum ALP, calcium-phosphorus product, and bone turnover markers rose significantly (Fig. 1). Furthermore, the proportions of parathyroid hyperplasia, osteoporosis, and vascular calcification were higher in the groups with iPTH 600–1499 pg/ml and iPTH 1500 pg/ml.

**Table 3:** Demographics and parameters of study patients according to iPTH level

Characteristics	Parathyroid hormone (pg/ml)			*p*
	iPTH 130–599 (*n* = 86)	iPTH 600–1499 (*n* = 82)	iPTH ≥ 1500 (*n* = 53)	
Age (year)	34.9 ± 13.8	50.2 ± 11.4**	46.6 ± 12.3**	< 0.001
Female (n, [%])	26 (30.2%)	44 (53.7%)*	26 (49.1%)*	0.006
BMI (kg/m^2^)	21.4 ± 3.4	22.1 ± 2.7	21.8 ± 3.0	0.4
Hemodialysis (n, [%])	53 (61.6%)	40 (48.8%)*	38 (71.7%)^#^	0.001
Dialysis duration (mo)	7.5 [2.5–28]	79.5 [52–119]**	89 [64–110]^**^	< 0.001
Hypertension (n, [%])	75 (87.2%)	76 (92.7%)	49 (92.5%)	0.4
Phosphate binders (n, [%])	34 (39.5%)	49 (59.8%)*	23 (43.4%)	0.024
Active vitamin D (n, [%])	26 (30.2%)	38 (46.3%)	22 (41.5%)	0.092
Calcimimetics (n, [%])	3 (3.5%)	24 (29.3%)**	11 (20.8%)*	< 0.001
Serum albumin (g/L)	43.2 ± 5.5	37.8 ± 4.7**	37.7 ± 4.7**	< 0.001
Hemoglobin (g/L)	110.1 ± 14.3	99.1 ± 16.3**	97.3 ± 16.9**	< 0.001
Serum calcium (mmol/L)	2.2 ± 0.2	2.3 ± 0.3	2.3 ± 0.2	0.051
Phosphates (mmol/L)	2.1 ± 0.6	2.1 ± 0.4	2.4 ± 0.5*^,^^#^	0.001
Ca × P product (mg^2^/dl^2^)	55.9 ± 16.9	62.8 ± 16.0*	71.1 ± 16.3**^,^^#^	< 0.001
sALP (U/L)	70 [56–96]	164 [112–214]**	268 [173–516]**^,^^##^	< 0.001
ß-CTX (pg/ml)	3105.5 [2055–4298]	5624 [4542–6001]**	6010 [6003–6023]**^,^^##^	< 0.001
P1NP(μg/ml)	334.4 [227.4–539]	1200 [1156–1203]**	1204 [1200–1208]**	< 0.001
OC (ng/ml)	237.4 [172–297.8]	289.8 [244.8–302]**	275.7 [214.7–302]*	< 0.001
25(OH)D (nmol/L)	44.7 [28.5–75.2]	41.3 [21.5–71.1]	44.8 [23.7–77.2]	0.3
Parathyroid hyperplasia (*n*, [%])	24 (27.9%)	77 (93.9%)**	53 (100.0%)**	< 0.001
Osteoporosis (*n*, [%])	10 (11.6%)	25 (30.5%)*	26 (49.1%)**^,^^#^	< 0.001
Vascular calcification (*n*, [%])	19 (22.1%)	54 (65.9%)**	40 (75.5%)**	< 0.001

*p < 0.05

**p < 0.001 vs. iPTH 200–600

^#^*p* < 0.05

^##^*p* < 0.001 vs. iPTH 600–1500

### Correlations of serum markers

Table 4 shows the results of univariate correlation analyses between bone turnover markers and demographic variables. The levels of the bone turnover marker osteocalcin, P1NP, and β-CTx correlated positively with age. Both formative and resorptive markers correlated positively with intact PTH, sALP and serum phosphate levels. Serum albumin and hemoglobin correlated negatively with bone turnover markers. There were no correlations between 25(OH)D and bone turnover markers. Serum calcium had a weak negative correlation with ß-CTX.

**Table 4:** Univariate correlations between bone turnover markers and characteristics of ESRD patients

	P1NP		OC		ß-CTX	
	rho		rho		rho	
Age, year	0.356	**	0.132	*	0.204	*
BMI, kg/m^2^	0.093		0.059		0.007	
Serum albumin, g/L	– 0.392	**	– 0.212	*	– 0.291	**
Hemoglobin, g/L	– 0.375	**	– 0.198	*	– 0.248	**
Serum calcium, mmol/L	0.111		0.065		0.216	*
Phosphates, mmol/L	0.142	*	0.193	*	0.319	**
Intact PTH, pg/mL	0.739	**	0.296	**	0.774	**
sALP, U/L	0.673	**	0.197	*	0.673	**
25(OH)D, nmol/L	– 0.102		– 0.042		0.010	

Data are Spearman’s rho with corresponding *p* values

*p < 0.05

**p < 0.001

## Discussion

Our study showed that osteoporosis and vascular calcification were two common complications in ESRD patients on transplant waiting lists. Vascular calcification was found in over 50% of the studied patients by plain radiography and echocardiogram. It has been suggested that low bone mass or volume is an independent predictor of coronary artery calcifications and even cardiovascular disease [14]. Our data confirmed an association between vascular calcification and osteoporosis in our patients. Based on clinical and experimental studies, the concept of a bone-vascular axis was established as complementary to the classic kidney-bone axis [10].

When evaluating the related factors in our study, vascular calcification and osteoporosis shared similar related risk factors, such as older age, female sex, longer dialysis duration, parathyroid hyperplasia and higher levels of iPTH, ALP, and bone turnover markers. According to our multivariate analyses, age and iPTH were independent risk predictors of both vascular calcification and osteoporosis. Several studies have shown that both bone loss and vascular calcification are age-related processes [15, 16]. Our results are in agreement with several studies that indicated that osteoporotic individuals were more likely to develop aortic calcification from various populations, including the advanced-CKD population. This possibility increased with aging [14, 17].

Moreover, after adjustment for age, iPTH was still an important risk factor for vascular calcification and osteoporosis in our ESRD patients. In patients with CKD/ESRD, secondary hyperparathyroidism could promote increased bone resorption and induce vascular calcification due to the endogenous release of phosphate and calcium. Based on epidemiological studies, for better outcomes, the Kidney Disease Improving Global Outcomes (KDIGO) guidelines recommend maintaining iPTH levels in the range of approximately 2 to 9 times the upper normal limit in patients with CKD G5D [1]. In a large international sample of patients on hemodialysis, Tentori F et al. reported that parathyroid hormone > 600 pg/ml was associated with a higher risk of cardiovascular mortality as well as all-cause and cardiovascular hospitalizations [18]. In addition, among individuals with stage 3 and 4 CKD, S. Geng et al. reported in a large population study using the Clinic Health System that PTH was an independent predictor of fractures, vascular events, and death [19].

In this study, we also discovered that moderate and severe hyperparathyroidism (iPTH 600–1499 pg/ml and iPTH 1500 pg/ml) were related to reduced levels of serum albumin and hemoglobin. Malnutrition is linked to an increase in morbidity and hospitalizations in patients with chronic renal disease and kidney transplants. Recent research indicated that individuals receiving maintenance hemodialysis for severe hyperparathyroidism had nutritional decline [20]. The involvement of high PTH levels in protein-energy loss highlighted the need of treating hyperparathyroidism early in patients awaiting kidney transplantation. Hypertension is a high prevalence complication in ESRD patients. A possible association between secondary hyperparathyroidism and hypertension has been reported [21]. However, the prevalence of hypertension was high in our study. The link between hypertension and secondary hyperthyroidism was not found to be significant.

Increased levels of both formative and resorptive markers were associated with decreased MBD and worsening parameters of bone quality. The International Federation of Clinical Chemistry and Laboratory Medicine (IFCC), in collaboration with the International Osteoporosis Foundation (IOF) and the National Bone Health Association (NBHA) in the US, have designated serum P1NP and serum ß-CTX in blood as reference standard bone turnover markers [22]. However, the diagnostic value of bone turnover markers is still controversial, especially in the advanced-CKD population. Coppolino et al. reported that ß-CTX had the potential ability to best estimate backward PTH into 12 months of interval in hemodialysis patients [23]. In our study, bone turnover markers were associated with osteoporosis and vascular calcification. However, the associations were attenuated when PTH levels were taken into account. Furthermore, bone turnover markers were positively correlated with the levels of PTH and ALP. The results supported that sustained elevation of PTH levels is associated with an abnormal phenotype of osteoblast function and subsequently a high turnover state in the bone, excess bone resorption, skeletal frailty and elevated fracture risk [24, 25]. More work is needed to discover the mechanism underlying the disturbances in the kidney–skeletal–cardiovascular axis.

Our study has several limitations. The small number of patients and the cross-sectional design may influence the power to assess the relationship between osteoporosis, vascular calcification and other factors. The lack of bone biopsy data made us unable to accurately diagnose the histological type of renal osteodystrophy. In addition, the patients in this study had high levels of iPTH, and a population with low bone turnover status may be uncovered.

## Conclusion

There is a high prevalence of vascular calcification and osteoporosis in ESRD patients awaiting transplantation. They shared similar risk factors, including older age, longer dialysis duration, parathyroid hyperplasia and higher levels of iPTH and bone turnover markers. In the multivariable regression model, age and iPTH were independent risk predictors of both vascular calcification and osteoporosis. Furthermore, the moderate and severe hyperparathyroidism were related with lower levels of serum albumin and hemoglobin. The involvement of high PTH levels in vascular calcification, osteoporosis and malnutrition indicated the need of treating hyperparathyroidism early in patients awaiting kidney transplantation. Bone turnover markers were positively correlated with the levels of PTH and ALP, but the diagnostic value of bone turnover markers has not been validated in ESRD patients. Prospective studies are needed to further examine the utility of bone turnover markers.

## Data Availability

The data underlying this article are available from the corresponding author on reasonable request.
